# Beyond BMI: Associations of Body-Shape Indices with CBC-Derived Systemic Inflammatory Indices in Adults with Metabolic Syndrome: A Cross-Sectional Study

**DOI:** 10.3390/metabo16100762

**Published:** 2026-10-10

**Authors:** Kamil Konur, Erol Karavar, Oğuzhan Kesen, Mümin Murat Yazıcı, Çağlayan Keklikkıran

**Affiliations:** 1 Department of Internal Medicine, Faculty of Medicine, Recep Tayyip Erdogan University, Rize 53020, Turkey; kamil.konur@erdogan.edu.tr (K.K.); erolkaravar@gmail.com (E.K.); 2 Division of Oncology, Department of Internal Medicine, Faculty of Medicine, Recep Tayyip Erdogan University, Rize 53020, Turkey; 3 Department of Emergency Medicine, Recep Tayyip Erdogan University Training and Research Hospital, Rize 53020, Turkey; mmuratyazici53@gmail.com; 4 Division of Gastroenterology, Department of Internal Medicine, Faculty of Medicine, Recep Tayyip Erdogan University, Rize 53020, Turkey; caglayan.keklikkiran@erdogan.edu.tr

**Keywords:** metabolic syndrome, weight-adjusted waist index, systemic inflammation response index

## Abstract

Background/Objectives: Metabolic syndrome (MetS) is characterized by central adiposity, metabolic dysregulation, and low-grade inflammation, but body mass index (BMI) may not fully capture body-shape variation in established MetS. We investigated whether body-shape indices were associated with complete blood count (CBC)–derived systemic inflammatory indices after accounting for BMI. Methods: In this cross-sectional study with prospective data collection, 331 adults with MetS were evaluated. Weight-adjusted waist index (WWI) was the primary anthropometric variable and systemic inflammation response index (SIRI) the primary inflammatory variable. Body roundness index (BRI), a body shape index (ABSI), conicity index, waist-to-height ratio (WHtR), and waist-to-hip ratio (WHR) were assessed as secondary anthropometric measures, together with the systemic immune-inflammation index (SII), neutrophil-to-lymphocyte ratio (NLR), platelet-to-lymphocyte ratio (PLR), and lymphocyte-to-monocyte ratio (LMR). Multivariable linear regression models were adjusted for age, sex, current smoking, and BMI. Results: WWI was not significantly associated with log-SIRI after adjustment for age, sex, current smoking, and BMI (standardized *β* = 0.067, 95% CI −0.052 to 0.185; *p* = 0.268). Adding WWI increased *R*^2^ from 0.091 to 0.094 (Δ*R*^2^ = 0.0034; partial *R*^2^ = 0.0038). None of the secondary anthropometric–inflammatory associations was statistically significant after false discovery rate (FDR) correction. Restricted cubic spline analysis showed no evidence of a nonlinear WWI–SIRI association (P-nonlinear = 0.582). Additional covariate analyses in which C-reactive protein, diabetes, estimated glomerular filtration rate, coronary artery disease and heart failure were added separately did not materially alter the WWI estimate. Conclusions: In adults with established MetS, we did not detect significant associations between WWI or other body-shape indices and CBC-derived systemic inflammatory indices after adjustment for BMI. These findings do not exclude smaller associations that may not have been detectable in this cohort.

## 1. Introduction

Metabolic syndrome (MetS) is a clinical condition characterized by the coexistence of abdominal obesity, impaired glucose metabolism, elevated blood pressure, and atherogenic dyslipidemia and is associated with an increased risk of cardiovascular disease and type 2 diabetes [1]. The prevalence of MetS has increased substantially over the past two decades. A large systematic review and modeling study covering the period from 2000 to 2023 estimated that approximately 1.54 billion adults worldwide were living with MetS in 2023 [2]. In MetS, abdominal and visceral adiposity, insulin resistance, adipose tissue dysfunction, and chronic low-grade inflammation are closely related features [1].

Although body mass index (BMI) is widely used to assess adiposity, it does not directly reflect body composition or fat distribution [3,4]. Individuals with the same BMI may differ, particularly in visceral adiposity and waist circumference [3,4]. Body-shape–based anthropometric indices such as weight-adjusted waist index (WWI), body roundness index (BRI), a body shape index (ABSI), and conicity index have been used to assess these differences [5,6,7]. Although associations of these indices with MetS and other cardiometabolic conditions have been reported in different populations, their performance relative to conventional anthropometric measures has been inconsistent [5,6,7]. For example, BRI has been associated with cardiovascular disease and mortality among individuals with MetS [8], whereas comparative studies evaluating the discrimination of MetS have shown that the performance of BRI, WWI, ABSI, and conventional anthropometric measures varies across populations [5,6,7]. Thus, the potential value of these indices within MetS may extend beyond their ability to discriminate the presence of MetS.

Systemic inflammation represents another source of heterogeneity within MetS. The systemic inflammation response index (SIRI), systemic immune-inflammation index (SII), neutrophil-to-lymphocyte ratio (NLR), platelet-to-lymphocyte ratio (PLR), and lymphocyte-to-monocyte ratio (LMR) are CBC-derived systemic inflammatory indices calculated from neutrophil, lymphocyte, monocyte, and platelet counts obtained from routine complete blood counts [9]. In a recent systematic review and meta-analysis, SII levels were higher in individuals with MetS; however, its ability to discriminate MetS was limited, and the area under the curve (AUC) estimate was based on only two studies [10]. Associations of SII and SIRI with obesity have also been reported in large population-based studies [11], while SIRI has been investigated in relation to various clinical outcomes across the cardiometabolic disease spectrum [12]. Recent studies directly examining the relationship between WWI and inflammation reported a positive correlation between WWI and SII in overweight and obese individuals [13], while another study based on National Health and Nutrition Examination Survey (NHANES) data found WWI to be positively associated with both SII and SIRI [14].

However, studies evaluating the relationship between WWI and CBC-derived systemic inflammatory indices have been conducted in the general population or among overweight and obese individuals [13,14]. Whether these associations also apply to individuals with established MetS, and particularly whether the WWI–SIRI association persists after adjustment for BMI, is less clear. Evaluating several body-shape indices against multiple CBC-derived systemic inflammatory indices within the same MetS population also allows assessment of whether observed associations are specific to a particular anthropometric or inflammatory index.

In this study, we aimed to evaluate the associations between body-shape indices and systemic inflammation in individuals with MetS. The primary analysis focused on the association between WWI and SIRI and whether this association persisted after adjustment for BMI. Associations between other body-shape indices and CBC-derived systemic inflammatory indices were evaluated as secondary analyses.

## 2. Materials and Methods

### 2.1. Study Design and Participants

This single-center, cross-sectional observational study prospectively collected data. The study was conducted at the Internal Medicine outpatient clinic of Recep Tayyip Erdoğan University Training and Research Hospital between December 2025 and July 2026. Participants were recruited using convenience sampling without consecutive enrollment. During the study period, adults attending the Internal Medicine outpatient clinic were considered for screening when the research team was available to perform the study-specific evaluation and obtain the required anthropometric measurements and blood samples. Eligible patients who met the MetS criteria, fulfilled the inclusion criteria, had none of the prespecified exclusion criteria, and provided written informed consent were enrolled. Because screening was not consecutive or based on a predefined sampling frame, not all potentially eligible patients attending the clinic during the study period were necessarily assessed for participation. A complete screening log was not maintained; therefore, the total number of patients approached or screened and the numbers excluded at each stage could not be reconstructed reliably. The final analytical sample comprised 331 participants who fulfilled the eligibility criteria and had complete and verifiable primary anthropometric and hematologic variables.

An a priori sample size calculation was performed during study planning using G*Power version 3.1. The calculation was based on a fixed-model multiple linear regression (*R*^2^ deviation from zero), assuming a medium effect size (Cohen’s *f*^2^ = 0.15), a two-sided *α* level of 0.05, 95% statistical power, and up to eight predictors. The minimum required sample size was approximately 160 participants. This calculation was intended as a general sample-size estimate for the planned multivariable analyses and was not specifically based on the incremental *R*^2^ attributable to WWI beyond the covariates in the primary model. No clinically meaningful incremental *R*^2^ threshold for WWI had been prespecified. To allow for incomplete or unverifiable data and the planned secondary analyses, a minimum recruitment target of 200 participants was set. This target represented a minimum rather than a fixed upper sample-size limit. Recruitment therefore continued throughout the prespecified study period from December 2025 to July 2026, and 331 participants constituted the final analytical sample after application of the eligibility criteria and data-quality procedures.

#### 2.1.1. Inclusion Criteria

Individuals aged ≥18 years who met the diagnostic criteria for metabolic syndrome according to the revised National Cholesterol Education Program Adult Treatment Panel III (NCEP-ATP III) criteria were eligible. For inclusion in the analysis, measurements of height, body weight, waist circumference, and hip circumference, as well as absolute neutrophil, lymphocyte, monocyte, and platelet counts, were required.

#### 2.1.2. Exclusion Criteria

Individuals were excluded if they had an acute infection at the time of assessment, a history of malignancy, autoimmune or connective tissue disease, current systemic corticosteroid or immunosuppressive therapy, or pregnancy.

For inclusion in the analytical dataset, complete primary anthropometric measurements and absolute neutrophil, lymphocyte, monocyte, and platelet counts were required. Participants with missing primary variables were excluded from the analysis. During data-quality review, values considered implausible or internally inconsistent were checked against the available source data. Measurements that could not be reconciled or confirmed after this review were considered unverifiable, and participants with unverifiable primary variables were not included in the final analytical dataset.

### 2.2. Definition of Metabolic Syndrome

Metabolic syndrome was defined according to the revised NCEP-ATP III criteria. MetS was diagnosed when at least three of the following five components were present: abdominal obesity, defined as waist circumference ≥ 102 cm in men or ≥88 cm in women; hypertriglyceridemia, defined as serum triglycerides ≥ 150 mg/dL or pharmacological treatment for elevated triglycerides; low HDL cholesterol, defined as HDL cholesterol < 40 mg/dL in men or <50 mg/dL in women or pharmacological treatment for low HDL cholesterol; elevated blood pressure, defined as systolic blood pressure ≥ 130 mmHg and/or diastolic blood pressure ≥ 85 mmHg or use of antihypertensive treatment; and the glycemic criterion, defined as fasting plasma glucose ≥ 100 mg/dL, a previous diagnosis of diabetes mellitus, or use of antidiabetic treatment. Glucose and triglyceride values obtained after at least 8 h of fasting were used to assess the laboratory-based MetS components. Participants meeting at least three of the five criteria were classified as having MetS.

### 2.3. Demographic and Clinical Assessment

Age, sex, smoking status, and exercise status were recorded. Smoking status was recorded as never, former, or current smoking for descriptive purposes. For regression analyses, smoking was dichotomized as current smoking versus non-current smoking, with former smokers included in the non-current smoking group. Physical activity was recorded descriptively as exercise on at least two days per week versus less frequent activity. Because this was a simple frequency-based measure rather than a quantitative assessment of physical activity, it was not included as a covariate in the primary regression model.

Comorbidities assessed included diabetes mellitus, heart failure, and coronary artery disease (CAD). CAD was defined as a history of myocardial infarction or known CAD/ischemic heart disease.

### 2.4. Anthropometric and Blood Pressure Measurements and Body-Shape Indices

Anthropometric assessment included standardized measurements of height, body weight, waist circumference, and hip circumference. Height was measured using a stadiometer with participants barefoot and standing upright, with the head positioned in the Frankfurt plane. Body weight was measured in kilograms using a calibrated digital scale while participants wore light clothing and no shoes.

Waist circumference was measured with the participant standing and the abdomen relaxed, at the level of the upper border of the iliac crest, at the end of normal expiration. A non-stretchable tape was kept parallel to the floor without compressing the skin. Hip circumference was measured at the widest circumference of the buttocks with the tape held parallel to the floor. All circumference measurements were recorded in centimeters.

Systolic and diastolic blood pressure were measured after participants had rested in a seated position for at least 5 min, using an automated sphygmomanometer with an appropriately sized cuff. Measurements were repeated twice after a short interval, and the mean of the two measurements was used in the analyses.

BMI was recalculated from verified height and body weight measurements rather than using previously calculated values stored in the database. To assess different aspects of body shape and abdominal adiposity, the WWI, BRI, ABSI, conicity index, WHtR, and WHR were calculated.

WWI was prespecified as the primary anthropometric variable. BRI, ABSI, and Conicity Index were evaluated as comparative body-shape indices, whereas WHtR and WHR were assessed as additional abdominal anthropometric measures. Because of the potential for high correlation and mathematical dependency, anthropometric indices were not entered simultaneously into the same regression model; each index was evaluated in a separate model using the same covariate set.

### 2.5. Laboratory Measurements and Systemic Inflammatory Indices

Blood samples were obtained on the same day as the anthropometric assessment after at least 8 h of fasting and were analyzed in the hospital central laboratory as part of the routine laboratory workflow. Complete blood counts were measured using a Mindray BC-6000 automated hematology analyzer (Mindray Bio-Medical Electronics Co., Ltd., Shenzhen, China). Serum CRP was measured on a Beckman Coulter AU680 clinical chemistry analyzer (Beckman Coulter, Brea, CA, USA) using a latex-based turbidimetric assay. The routine, standard CRP application was used rather than a high-sensitivity CRP assay.

Before calculation of the CBC-derived inflammatory indices, hematologic data were checked for missing values, unit inconsistencies, and implausible or internally inconsistent results. Flagged values were reviewed against the available source laboratory records. Values that could not be confirmed or reconciled were considered unverifiable. Hematologic abnormalities such as leukocytosis, neutropenia, thrombocytopenia, or anemia were not themselves prespecified exclusion criteria. No unresolved biologically implausible or extreme hematologic values remained in the final analytical dataset.

Absolute neutrophil, lymphocyte, monocyte, and platelet counts were used to calculate the CBC-derived systemic inflammatory indices. The SIRI, SII, neutrophil-to-lymphocyte ratio (NLR), platelet-to-lymphocyte ratio (PLR), and lymphocyte-to-monocyte ratio (LMR) were derived from complete blood count parameters.

SIRI was prespecified as the primary CBC-derived systemic inflammatory index. SII was considered the main secondary index, while NLR, PLR, and LMR were evaluated as additional CBC-derived systemic inflammatory indices. CRP was evaluated separately in additional covariate analyses as a systemic inflammatory marker distinct from the CBC-derived indices. SIRI, SII, NLR, PLR, and LMR were considered indirect systemic inflammatory indices derived from circulating blood-cell counts. These measures do not directly assess cytokine activity, adipose tissue inflammation, or tissue-level inflammatory processes.

The formulas used to calculate the anthropometric and CBC-derived systemic inflammatory indices are provided in Supplementary Table S1.

### 2.6. Primary and Secondary Analysis Framework

WWI was selected a priori as the primary anthropometric variable because it incorporates waist circumference relative to body weight and has been proposed as a marker of body-shape variation beyond BMI. Recent studies have also reported associations of WWI with CRP and CBC-derived inflammatory indices, including SII and SIRI [13,14,15], providing a specific rationale for evaluating WWI–SIRI as the primary association. SIRI was selected a priori as the primary inflammatory variable because it integrates neutrophil, monocyte, and lymphocyte counts and had previously been examined in relation to both obesity-related phenotypes and WWI [11,14]. SII, NLR, PLR, and LMR were retained as secondary inflammatory indices to assess whether findings were consistent across alternative CBC-derived measures. The primary analysis therefore evaluated the association between WWI and SIRI and whether this association persisted after adjustment for age, sex, current smoking, and BMI.

Covariates for the primary model were selected a priori based on their potential relationships with both anthropometric characteristics and systemic inflammatory indices rather than on univariable statistical significance. Age and sex were included because both body composition and circulating leukocyte profiles vary according to these characteristics.

Smoking status was recorded as never, former, or current smoking. For the regression analyses, smoking was dichotomized as current versus non-current smoking, with former smokers included in the non-current group. This coding was used to distinguish active smoking exposure from the absence of current smoking. Information on cumulative smoking exposure, smoking duration, and time since cessation was not available; therefore, residual effects of previous smoking exposure could not be accounted for.

BMI was included specifically because the primary research question was whether WWI was associated with SIRI beyond overall adiposity as represented by BMI.

Because BMI and the examined anthropometric indices share underlying components such as body weight, height, and waist circumference, adjustment for BMI could attenuate associations and increase collinearity. BMI was nevertheless retained in the primary model because the prespecified research question concerned the association of WWI with SIRI beyond overall adiposity as represented by BMI. To assess the influence of this adjustment, we also evaluated models without BMI and compared the resulting estimates. Anthropometric indices were entered in separate models rather than simultaneously to avoid additional collinearity among mathematically related measures.

Additional clinical variables, including diabetes mellitus, eGFR, CAD, heart failure, and CRP, were examined separately in additional covariate analyses rather than incorporated into the primary model. These variables were not treated as mandatory confounders because some may represent downstream clinical manifestations of the metabolic phenotype or related inflammatory processes, and their inclusion in the primary model could introduce overadjustment. Secondary analyses evaluated the associations of BRI, ABSI, Conicity Index, WHtR, and WHR with SIRI. Associations between the body-shape indices and SII, NLR, PLR, and LMR were also examined. The multiple-testing burden in the secondary analyses was addressed using Benjamini–Hochberg false discovery rate (FDR) correction across the anthropometric indices within each inflammatory outcome.

### 2.7. Ethical Approval

The study protocol was approved by the Recep Tayyip Erdogan University Non-Interventional Clinical Research Ethics Committee on 26 September 2025 (decision no. 2025/385). The study was conducted in accordance with the principles of the Declaration of Helsinki, and written informed consent was obtained from all participants before enrollment.

### 2.8. Statistical Analysis

Statistical analyses were performed using jamovi software (version 1.6, The jamovi Project, Sydney, Australia). Restricted cubic spline analyses were conducted using RStudio (version 2024.12.1+563). Categorical variables are presented as counts and percentages, and continuous variables as mean ± standard deviation or median (IQR), according to their distributions. Distributional characteristics were assessed using visual methods and the Shapiro–Wilk test.

Because SIRI, SII, NLR, PLR, LMR, and CRP showed right-skewed distributions, natural logarithmic transformation was applied before regression analysis to improve model fit and residual behavior. This transformation addressed the distribution of the outcome variables and did not assume a linear exposure–outcome relationship. Potential non-linearity in the WWI–log-SIRI association was evaluated separately using restricted cubic spline regression. Anthropometric indices were standardized to z-scores. Associations were evaluated using multivariable linear regression models. For models using z-standardized anthropometric indices, results are reported as unstandardized B coefficients representing the change in the log-transformed inflammatory outcome per 1-SD increase in the anthropometric index, with 95% confidence intervals. For the hierarchical primary model, both unstandardized B and standardized β coefficients are reported.

In the primary analysis, the association between WWI and log-SIRI was adjusted for age, sex, current smoking status, and BMI. The change in *R*^2^ and partial *R*^2^ associated with the addition of WWI to the model were calculated. Other anthropometric indices were evaluated in separate models using the same covariate set; highly correlated or mathematically related indices were not entered simultaneously into the same model. Potential non-linearity in the WWI–log-SIRI association was examined using restricted cubic spline regression in R with the rms package. An ordinary least-squares model was fitted using the ols() function with the spline term specified as rcs(WWI, 3). Three knots were placed at the 10th, 50th, and 90th percentiles of the WWI distribution, with the median WWI value (11.7) used as the reference. The spline model was adjusted for age, sex, current smoking, and BMI, consistent with the primary linear model. Adjusted estimates with 95% confidence intervals were generated using the Predict() function, and the overall association and non-linear component were tested separately.

The prespecified WWI–SIRI association constituted the single primary hypothesis and was evaluated without adjustment for multiple comparisons. All other anthropometric–inflammatory associations were considered secondary. For the secondary analyses, each inflammatory outcome was treated as a separate hypothesis family, and the Benjamini–Hochberg false discovery rate (FDR) procedure was applied across the anthropometric indices tested within that outcome. Thus, the resulting q-values controlled the FDR within each outcome-specific family rather than across all secondary analyses collectively. An FDR-adjusted *q* < 0.05 was considered statistically significant. Additional covariate analyses for the primary WWI–SIRI association were performed by separately adding CRP, diabetes mellitus, eGFR, CAD, and heart failure to the primary model. These analyses were intended to assess the influence of each variable individually and were not considered a comprehensive assessment of joint clinical confounding.

Regression diagnostics were assessed using residual-versus-fitted and normal Q–Q plots. Multicollinearity was evaluated using variance inflation factors (VIFs) and tolerance values, and potentially influential observations were assessed using standardized residuals and Cook’s distance. Heteroscedasticity was additionally assessed using the Breusch–Pagan test, and residual autocorrelation using the Durbin–Watson statistic. Diagnostic results are provided in the Supplementary Table S2 and Supplementary Figure S1.

No imputation was performed for missing data. A two-sided *p* < 0.05 was considered statistically significant for the primary analyses, whereas an FDR-adjusted *q* < 0.05 was used as the significance threshold for secondary analyses.

## 3. Results

### 3.1. Baseline Characteristics of the Study Population

The final analytical population comprised 331 participants. Of these, 210 (63.4%) were women and 121 (36.6%) were men, and the median age was 59 years (IQR: 50–68). Diabetes mellitus was present in 231 participants (69.8%), CAD in 60 (18.1%), and heart failure in 10 (3.0%). Current smoking was reported by 24.5% of participants, and 51.1% reported exercising at least two days per week.

The median BMI was 33.6 kg/m^2^ (IQR: 29.8–38.3), waist circumference was 109 cm (IQR: 100–120), and hip circumference was 116 cm (IQR: 108–125). The mean WWI was 11.7 ± 0.92 and the mean ABSI was 0.0825 ± 0.0069. Median BRI was 7.16 (IQR: 5.85–8.86), WHtR was 0.67 (IQR: 0.62–0.74), and WHR was 0.94 (IQR: 0.90–0.99).

Median glucose was 119 mg/dL (IQR: 100–155), triglycerides 163 mg/dL (IQR: 116–224), HDL cholesterol 48 mg/dL (IQR: 40–57.7), and CRP 3.84 mg/L (IQR: 1.94–8.00). Median SIRI was 0.75 (IQR: 0.56–1.14), SII 469 (IQR: 355–607), NLR 1.73 (IQR: 1.38–2.37), PLR 116 (IQR: 94.2–148), and LMR 5.12 (IQR: 4.12–6.32) (Table 1).

### 3.2. Primary Association Between WWI and SIRI

The base model including age, sex, current smoking, and BMI explained 9.1% of the variance in log-SIRI (*R*^2^ = 0.091; adjusted *R*^2^ = 0.077). After WWI was added to the model, it was not significantly associated with log-SIRI (standardized *β* = 0.067, 95% CI −0.052 to 0.185; *p* = 0.268).

The addition of WWI increased *R*^2^ from 0.091 to 0.094 (Δ*R*^2^ = 0.0034; *F*-change = 1.229; *p* = 0.268), corresponding to a partial *R*^2^ of 0.0038. The adjusted *R*^2^ remained 0.077 (Table 2).

### 3.3. Secondary Analyses of Other Anthropometric Indices with SIRI

As secondary analyses, the associations of ABSI, BRI, conicity index, WHtR, and WHR with log-SIRI were evaluated using the same covariate structure as the primary model. In models adjusted for age, sex, and current smoking status, none of WWI, ABSI, BRI, conicity index, WHtR, WHR, or BMI was significantly associated with log-SIRI.

**Figure 1:**
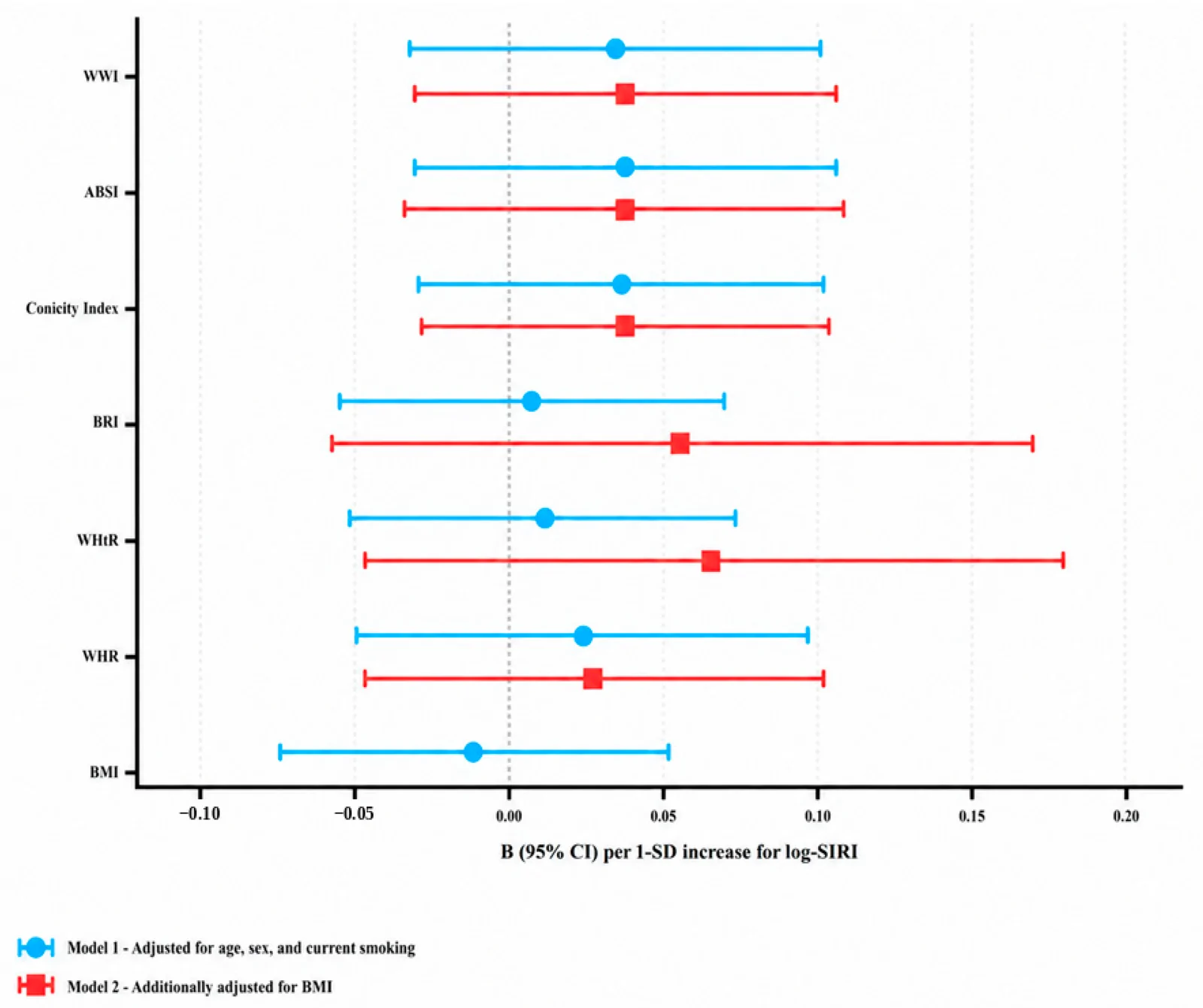
Adjusted associations of anthropometric indices with log-SIRI. SIRI: Systemic Inflammation Response Index, WWI: Weight-adjusted Waist Index, ABSI: A Body Shape Index, BRI: Body Roundness Index, WHtR: Waist-to-Height Ratio, WHR: Waist-to-Hip Ratio, BMI: Body Mass Index, CI: Confidence Interval. The vertical dashed line indicates the null value (B = 0).

Results were similar when BMI was additionally included as a covariate in models evaluating WWI, ABSI, BRI, conicity index, WHtR, and WHR. Regression coefficients per 1-standard-deviation increase ranged from 0.028 to 0.067; all 95% confidence intervals included zero, and all *p* values were >0.20. The partial *R*^2^ for each index was ≤0.004 (Table 3). None of the secondary associations with SIRI was statistically significant, either before or after FDR correction (all *q* ≥ 0.408). Coefficients and 95% confidence intervals from both adjustment models are shown in Figure 1.

### 3.4. Secondary Analyses with Other CBC-Derived Inflammatory Indices

In secondary analyses using SII, NLR, PLR, and LMR as outcomes, none of the anthropometric indices was significantly associated with the CBC-derived inflammatory indices after adjustment for age, sex, current smoking, and BMI. The results remained non-significant after Benjamini–Hochberg FDR correction, with q values ≥ 0.188 for all evaluated associations (Table 4).

### 3.5. Additional Covariate and Interaction Analyses

When log-CRP was added to the primary model, the regression coefficient for WWI was 0.025 (95% CI: −0.042 to 0.092; *p* = 0.466). When diabetes mellitus, eGFR, CAD, and heart failure were added separately to the model, the WWI coefficients remained between 0.038 and 0.039, with no statistically significant association in any model (all *p* ≥ 0.261).

The WWI × sex (*B* = 0.029; 95% CI: −0.119 to 0.177; *p* = 0.699) interaction terms were also not statistically significant (Table 5).

### 3.6. Assessment of a Nonlinear Association Between WWI and SIRI

**Figure 2:**
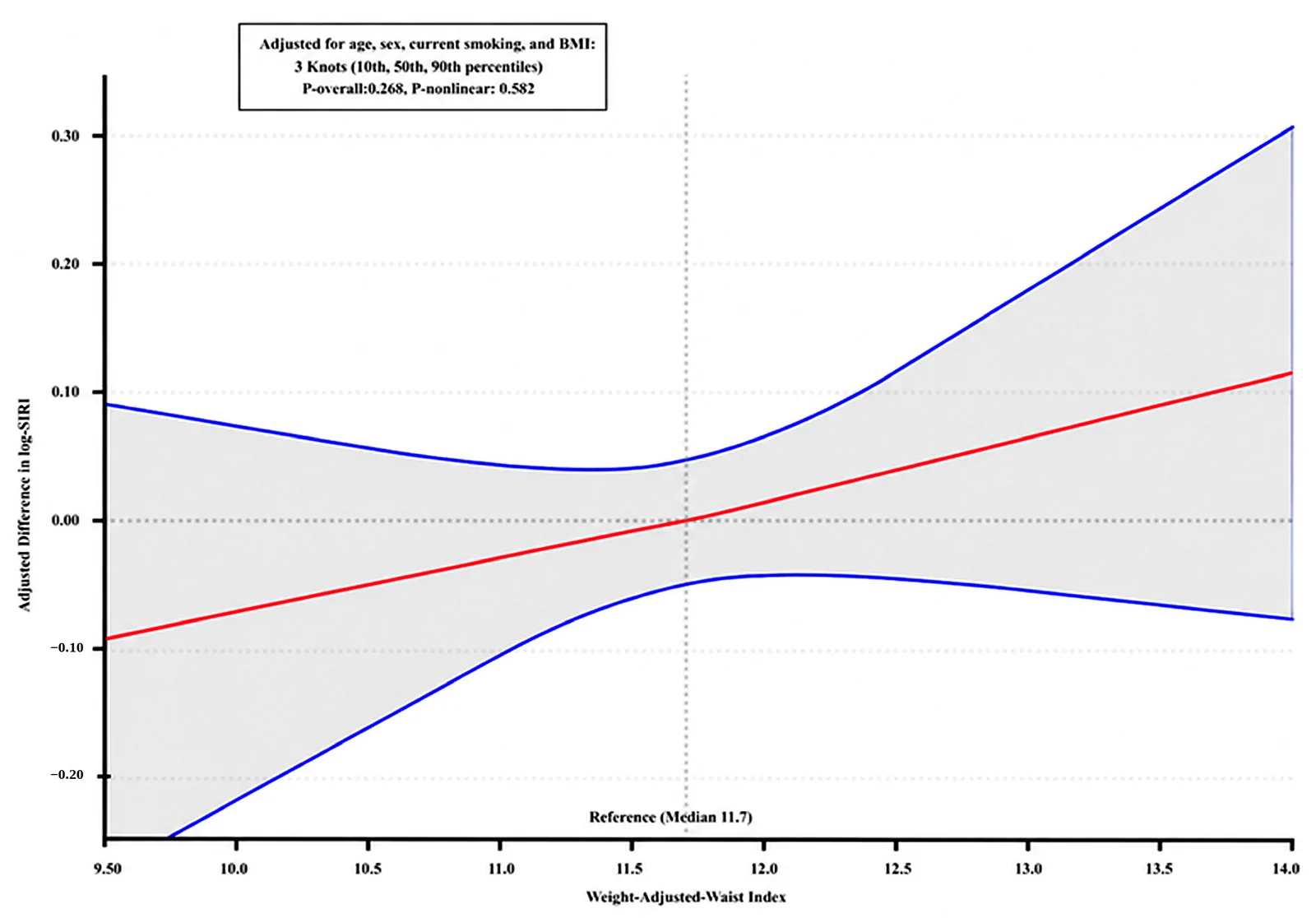
Restricted cubic spline analysis of the association between WWI and log-SIRI. The model was adjusted for age, sex, current smoking, and BMI. Three knots were placed at the 10th, 50th, and 90th percentiles of WWI, with the median WWI value (11.7) used as the reference. The solid line represents the adjusted estimate and the shaded area the 95% confidence interval. *p*-overall = 0.268; *p*-nonlinear = 0.582.

Restricted cubic spline analysis provided no evidence of a nonlinear association between WWI and log-SIRI after adjustment for age, sex, current smoking, and BMI. The overall association was not statistically significant (*p*-overall = 0.268), and the test for nonlinearity was also non-significant (*p*-nonlinear = 0.582) (Figure 2).

## 4. Discussion

The primary analysis showed no statistically significant association between WWI and SIRI after adjustment for age, sex, current smoking, and BMI. Secondary analyses showed a similar pattern across the other anthropometric and CBC-derived inflammatory indices. Because FDR correction was applied within outcome-specific families rather than across all secondary tests collectively, these findings should be interpreted as supportive rather than confirmatory and not as equivalent to the prespecified WWI–SIRI comparison.

Adjustment for BMI may attenuate associations involving body-shape indices because these measures share information on body size and adiposity. In the present analysis, however, the WWI–SIRI estimate changed little after BMI was added, and the association was also non-significant in the model without BMI. Thus, the non-significant primary finding was not attributable solely to BMI adjustment. Because the study is cross-sectional, the adjusted estimates should be interpreted as conditional associations rather than causal effects. BMI may also partly reflect pathways linking body shape and inflammation; therefore, adjustment could remove part of the association of interest in addition to controlling confounding.

Previous population-based studies have reported positive associations between WWI and inflammatory markers, including CRP, SII, and SIRI [13,14,15], while other analyses have linked obesity or visceral adiposity with higher CBC-derived inflammatory indices [11,16]. These studies included broader populations, whereas our cohort was restricted to individuals with established MetS. Because MetS is defined partly by waist circumference and several metabolic characteristics, restricting the analysis to this phenotype may narrow the distributions of anthropometric and metabolic variables and attenuate observable associations. Conditioning on a composite MetS phenotype could also introduce selection-related bias if the characteristics defining MetS are related to both adiposity and inflammation. Whether such bias occurred, and its direction or magnitude, cannot be determined from the present data. The restricted cubic spline analysis likewise provided no evidence of a statistically detectable nonlinear WWI–SIRI association.

The literature on CBC-derived inflammatory indices also suggests that the presence of MetS and inflammatory heterogeneity within established MetS should be considered as distinct questions. In the MESA cohort, higher SII was associated with the presence of MetS and several MetS components [17]. Similarly, Marra et al. reported an association between SIRI and MetS in adults with severe obesity, although its discriminatory performance was limited [18]. These studies support the presence of inflammatory differences between individuals with and without MetS, but they do not establish that variation in body shape among individuals who already have MetS explains SIRI or SII levels. Our research question addresses this latter issue.

The absence of detectable associations with CBC-derived inflammatory indices is not necessarily inconsistent with studies linking anthropometric indices to clinical outcomes. WWI and BRI have been associated with cardiovascular disease and mortality in populations with MetS [19,20], and a recent meta-analysis reported associations between BRI and cardiovascular disease and diabetes [21]. Wang et al. also reported that several inflammatory indices statistically mediated the association between WWI and mortality [22]. These studies, however, focused on long-term clinical outcomes, whereas our analysis examined cross-sectional associations between anthropometric and hematologic measures. An anthropometric index may therefore be associated with clinical outcomes without showing a detectable cross-sectional association with CBC-derived inflammatory indices. CBC-derived inflammatory indices may also provide prognostic information independently of anthropometric measures; in a recent prospective cohort of older emergency department patients, SII remained independently associated with 30-day mortality after adjustment for comorbidity burden and frailty [23].

Anthropometric indices and CBC-derived inflammatory indices capture different aspects of the metabolic phenotype. Visceral and ectopic adiposity have been linked to adipose tissue dysfunction and inflammatory signaling [24,25], but these processes were not assessed in our study. WWI, BRI, and ABSI are indirect anthropometric measures, whereas SIRI and SII are derived from circulating blood-cell counts and do not specifically reflect adipose tissue inflammation. The non-significant cross-sectional associations may therefore partly reflect differences in what these measures capture, although this remains a possible interpretation rather than a mechanism demonstrated by our data.

When diabetes mellitus, eGFR, CAD, heart failure, and CRP were added separately to the primary model, the WWI estimate changed little. These analyses indicate that the primary estimate was not materially altered by any one of these variables individually. However, because the variables were not modeled jointly, these analyses do not establish robustness to combined clinical confounding and cannot exclude residual confounding.

This study has several limitations. Its cross-sectional design does not allow assessment of the temporal direction between body shape and inflammation. The single-center MetS cohort was recruited using convenience rather than consecutive sampling. Because participation depended partly on the availability of the research team and the feasibility of completing the study assessment, the enrolled sample may differ from potentially eligible patients who attended the clinic but were not screened or enrolled. This may have introduced selection bias and limits the generalizability of the observed associations to the broader population of individuals with MetS. The direction and magnitude of this potential bias cannot be determined from the available data. A fundamental design limitation is that all participants were selected on the basis of established MetS. Because MetS is partly defined by waist circumference and several metabolic characteristics, restricting the sample to this phenotype may have narrowed the distributions of anthropometric and metabolic variables and reduced between-participant contrast. Such restriction of range may attenuate detectable anthropometry–inflammation associations. In addition, conditioning on MetS, a composite phenotype related to both adiposity and metabolic abnormalities, may introduce selection or conditioning bias and alter associations observed within the selected cohort. The direction and magnitude of these effects cannot be quantified without a comparison group outside the MetS population. Inflammatory assessment was limited to single-time-point measurements of CBC-derived indices and CRP. SIRI, SII, NLR, PLR, and LMR are derived from circulating blood-cell counts and may not adequately reflect tissue-level or adipose-specific inflammation. Cytokines such as IL-6 and TNF-α, oxidative stress markers, and direct measures of adipose tissue inflammation were not assessed. Visceral adipose tissue was also not assessed directly using imaging or body-composition methods. The additional clinical covariates were evaluated individually rather than jointly; therefore, their combined confounding effect was not assessed. Former smokers were grouped with never smokers in the regression analyses, and information on cumulative tobacco exposure and time since smoking cessation was unavailable. Residual confounding related to previous smoking exposure therefore cannot be excluded. Physical activity was assessed only using a simple frequency-based classification without information on duration, intensity, exercise type, or total weekly activity. Because this measure was not included in the multivariable models, residual confounding related to physical activity cannot be excluded. Although individuals receiving systemic corticosteroids or immunosuppressive therapy were excluded, detailed exposure to glucose-lowering, lipid-lowering, antihypertensive, and other cardiovascular medications was not modeled. Residual confounding related to treatment patterns therefore cannot be excluded. The a priori sample size calculation was based on the explanatory capacity of the overall multivariable model rather than the incremental R^2^ attributable specifically to WWI. The study may therefore have had limited power to detect small incremental WWI effects, and the non-significant primary association should not be interpreted as demonstrating equivalence or absence of an association. The exclusion of participants with missing key variables under the complete-case approach may also have introduced selection bias.

The prespecification of the primary WWI–SIRI association, avoidance of simultaneously entering multiple anthropometric indices into the same model, use of FDR correction for secondary analyses, and assessment of the association using spline and additional covariate analyses were strengths of the analytical framework. However, the absence of statistically significant associations should not be interpreted as evidence that body shape and systemic inflammation are unrelated. The confidence intervals do not completely exclude small associations, and the present study was not designed to establish equivalence or absence of an effect. The findings therefore indicate that we did not detect associations of the magnitude assessed in this cohort rather than demonstrating that no relationship exists.

The non-significant findings may reflect restricted anthropometric variability within MetS, differences in what anthropometric and CBC-derived inflammatory indices capture, or associations smaller than those detectable in this sample. Studies combining imaging-based assessment of visceral adiposity with repeated measurements of cytokines and CBC-derived inflammatory indices could help distinguish among these possibilities.

## 5. Conclusions

Among individuals with established metabolic syndrome, neither WWI nor the other body-shape indices examined were significantly associated with SIRI or the other CBC-derived inflammatory indices. The WWI–SIRI association remained non-significant after adjustment for age, sex, current smoking, and BMI, with similar results across the secondary analyses. These findings should not be interpreted as evidence that body shape and inflammation are unrelated, because small associations cannot be excluded. Rather, the present study did not detect statistically significant associations between the anthropometric and CBC-derived inflammatory indices examined in this MetS cohort.

## Figures and Tables

**Table 1:** Study sample characteristics.

Characteristics	Total Sample (*n* = 331)
Sex	
Male, *n* (%)	121 (36.6)
Female, *n* (%)	210 (63.4)
Age (years), median (IQR)	59 (50–68)
Comorbidities	
Diabetes, *n* (%)	231 (69.8)
CAD, *n* (%)	60 (18.1)
Congestive heart failure, *n* (%)	10 (3.0)
Smoking status	
Has never smoked, *n* (%)	219 (66.1)
Former smoker, *n* (%)	31 (9.4)
Currently smokes, *n* (%)	81 (24.5)
Exercise	
Two days or more a week, *n* (%)	169 (51.1)
Less than two days a week, *n* (%)	162 (48.9)
Height (centimetre), median (IQR)	162 (155–170)
Weight (kilogram), median (IQR)	90 (79–100)
Body Mass Index (kg/m^2^), median (IQR)	33.6 (29.8–38.3)
Waist circumference (centimetre), median (IQR)	109 (100–120)
Hip circumference (centimetre), median (IQR)	116 (108–125)
Laboratory Indices	
White Blood Cell (10^3^/uL), median (IQR)	7.13 (6.01–8.57)
Lymphocyte (10^3^/uL), median (IQR)	2.30 (1.82–2.92)
Neutrophil (10^3^/uL), median (IQR)	4.03 (3.31–5.16)
Monocyte (10^3^/uL), median (IQR)	0.46 (0.36–0.57)
Platelet (10^3^/uL), median (IQR)	273 (225–319)
Glucose (mg/dL), median (IQR)	119 (100–155)
Creatinine (mg/dL), median (IQR)	0.83 (0.68–1.03)
Glomerular Filtration Rate (mL/min/1.73 m^2^), median (IQR)	87 (69–100)
Triglyceride (mg/dL), median (IQR)	163 (116–224)
High-Density Lipoprotein Cholesterol (mg/dL), median (IQR)	48 (40–57.7)
C-reactive protein (mg/L), median (IQR)	3.84 (1.94–8.00)
Variables	
Neutrophil-to-Lymphocyte Ratio (NLR), median (IQR)	1.73 (1.38–2.37)
Platelet-to-Lymphocyte Ratio (PLR), median (IQR)	116 (94.2–148)
Lymphocyte-to-Monocyte Ratio (LMR), median (IQR)	5.12 (4.12–6.32)
Systemic Immune-Inflammation Index (SII), median (IQR)	469 (355–607)
Systemic Inflammation Response Index (SIRI), median (IQR)	0.75 (0.56–1.14)
Waist-to-Hip Ratio (WHR), median (IQR)	0.94 (0.90–0.99)
Waist-to-Height Ratio (WHtR), median (IQR)	0.67 (0.62–0.74)
Conicity Index, mean ± SD	1.36 ± 0.10
Body Roundness Index (BRI), median (IQR)	7.16 (5.85–8.86)
A Body Shape Index (ABSI), mean ± SD	0.0825 ± 0.0069
Weight-adjusted Waist Index (WWI), mean ± SD	11.7 ± 0.92

IQR, interquartile range (25th–75th percentile); SD, standard deviation.

**Table 2:** Hierarchical linear regression models for log-SIRI before and after addition of WWI.

Variable	Unstandardized B (95% CI)	Standardized *β* (95% CI)	*p* Value
Model 1			
Age	0.008 (0.004–0.013)	0.193 (0.083–0.304)	0.001
Sex	0.129 (−0.006–0.264)	0.222 (−0.011–0.455)	0.061
Current smoking *	0.314 (0.165–0.463)	0.542 (0.284–0.799)	0.001
BMI	−0.002 (−0.011–0.008)	−0.019 (−0.128–0.091)	0.739
Model 2			
Age	0.007 (0.002–0.012)	0.164 (0.041–0.286)	0.009
Sex	0.137 (0.001–0.272)	0.236 (0.002–0.470)	0.048
Current smoking *	0.313 (0.164–0.462)	0.540 (0.282–0.797)	0.001
BMI	−0.003 (−0.013–0.007)	−0.031 (−0.143–0.081)	0.586
WWI	0.042 (−0.033–0.117)	0.067 (−0.052–0.185)	0.268

Note: Model 1 included age, sex, current smoking, and BMI. Model 2 additionally included WWI. Model 1: R^2^ = 0.091; adjusted R^2^ = 0.077. Model 2: R^2^ = 0.094; adjusted R^2^ = 0.077. Addition of WWI: ΔR^2^ = 0.0034; partial R^2^ = 0.0038; F-change = 1.229; *p* = 0.268. SIRI, systemic inflammation response index; BMI, body mass index; WWI, weight-adjusted waist index. * Current smoking was coded as current versus non-current smoking, with former smokers included in the non-current group.

**Table 3:** Adjusted Associations of Anthropometric Indices with log-SIRI.

Anthropometric Index	B (95% CI) per 1-SD Increase	*p* Value	FDR *q*	Partial *R*^2^
Model 1				
WWI	0.035 (−0.032–0.102)	0.307	—	0.003
ABSI	0.038 (−0.030–0.107)	0.269	—	0.004
BRI	0.008 (−0.054–0.071)	0.792	—	0.001
Conicity Index	0.037 (−0.029–0.103)	0.269	—	0.004
WHtR	0.012 (−0.051–0.075)	0.715	—	0.001
WHR	0.025 (−0.048–0.098)	0.506	—	0.001
BMI	−0.011 (−0.074–0.053)	0.739	—	0.001
Model 2				
WWI	0.039 (−0.030–0.107)	0.268	—	0.004
ABSI	0.038 (−0.033–0.110)	0.292	0.408	0.003
BRI	0.057 (−0.057–0.171)	0.326	0.408	0.003
Conicity Index	0.038 (−0.028–0.105)	0.253	0.408	0.004
WHtR	0.067 (−0.046–0.181)	0.246	0.408	0.004
WHR	0.028 (−0.046–0.103)	0.456	0.456	0.002

Note 1: All anthropometric indices were converted to standard deviation scores (z-scores); coefficients reflect the change in log-SIRI per 1 SD increase in each index. Note 2: Model 1 is adjusted for age, sex, and current smoking status. Model 2 is additionally adjusted for BMI. Note 3: The prespecified WWI–SIRI association in Model 2 constituted the primary comparison and was not included in the multiple-comparison correction. Benjamini–Hochberg FDR correction was applied to the secondary anthropometric indices in Model 2. An FDR-adjusted q < 0.05 was considered statistically significant. Note 4: Model 1 is presented to allow comparison of the estimates before and after adjustment for BMI and was not treated as a separate family of secondary hypothesis tests. Note 5: Partial *R*^2^ represents the proportion of residual variance uniquely explained by the respective anthropometric index. WWI: Weight-adjusted Waist Index, SIRI: Systemic Inflammation Response Index, ABSI: A Body Shape Index, BRI: Body Roundness Index, WHtR: Waist-to-Height Ratio, WHR: Waist-to-Hip Ratio, BMI: Body Mass Index.

**Table 4:** Multivariable Associations Between Anthropometric Indices and Secondary CBC-Derived Systemic Inflammatory Indices with False Discovery Rate Adjustment.

Anthropometric Index	Log-SII *B* (95% CI) per 1-SD Increase	Raw *p*	FDR *q*
WWI	0.019 (−0.044–0.082)	0.554	0.835
ABSI	0.013 (−0.053–0.079)	0.697	0.835
BRI	0.041 (−0.064–0.145)	0.445	0.835
Conicity Index	0.011 (−0.050–0.072)	0.716	0.835
WHtR	0.031 (−0.074–0.136)	0.560	0.835
WHR	−0.001 (−0.069–0.068)	0.986	0.986
Anthropometric Index	Log-NLR *B* (95% CI) per 1-SD increase	Raw *p*	FDR *q*
WWI	0.001 (−0.049–0.052)	0.960	0.960
ABSI	−0.005 (−0.058–0.047)	0.844	0.960
BRI	0.017 (−0.067–0.101)	0.689	0.960
Conicity Index	−0.003 (−0.051–0.046)	0.913	0.960
WHtR	0.009 (−0.074–0.093)	0.829	0.960
WHR	0.021 (−0.034–0.075)	0.459	0.960
Anthropometric Index	Log-PLR *B* (95% CI) per 1-SD increase	Raw *p*	FDR *q*
WWI	−0.031 (−0.076–0.013)	0.161	0.188
ABSI	−0.040 (−0.086–0.006)	0.091	0.188
BRI	−0.037 (−0.110–0.036)	0.323	0.323
Conicity Index	−0.040 (−0.082–0.003)	0.067	0.188
WHtR	−0.056 (−0.129–0.017)	0.133	0.188
WHR	−0.035 (−0.082–0.013)	0.154	0.188
Anthropometric Index	Log-LMR *B* (95% CI) per 1-SD increase	Raw *p*	FDR *q*
WWI	0.012 (−0.031–0.055)	0.589	0.807
ABSI	0.014 (−0.031–0.059)	0.531	0.807
BRI	0.021 (−0.051–0.092)	0.570	0.807
Conicity Index	0.012 (−0.029–0.054)	0.554	0.807
WHtR	0.020 (−0.051–0.091)	0.585	0.807
WHR	0.006 (−0.041–0.052)	0.807	0.807

Note 1: Anthropometric indices are evaluated per 1 SD increase. Benjamini–Hochberg FDR correction was applied across indices within each inflammatory outcome (q-value threshold < 0.05). Note 2: All models were adjusted for age, sex, current smoking status, and BMI. WWI: Weight-adjusted Waist Index, ABSI: A Body Shape Index, BRI: Body Roundness Index, WHtR: Waist-to-Height Ratio, WHR: Waist-to-Hip Ratio, BMI: Body Mass Index, SII: Systemic Immune-Inflammation Index, NLR: Neutrophil-to-Lymphocyte Ratio, PLR: Platelet-to-Lymphocyte Ratio, LMR: Lymphocyte-to-Monocyte Ratio.

**Table 5:** Additional Covariate and Interaction Analyses of the Association Between WWI and log-SIRI.

Model Specification	Parameter Evaluated	B (95% CI) per 1-SD Increase	*T* Value	*p* Value
Additional Covariate Analyses				
Primary Multivariable Model	WWI (per 1 SD)	0.039 (−0.030–0.107)	1.109	0.268
+Adjustment for log-CRP	WWI (per 1 SD)	0.025 (−0.042–0.092)	0.730	0.466
+Adjustment for DM	WWI (per 1 SD)	0.039 (−0.030–0.108)	1.103	0.271
+Adjustment for eGFR	WWI (per 1 SD)	0.039 (−0.029–0.107)	1.127	0.261
+Adjustment for CAD	WWI (per 1 SD)	0.038 (−0.031–0.107)	1.085	0.279
+Adjustment for CHF	WWI (per 1 SD)	0.038 (−0.031–0.107)	1.092	0.276
Interaction Analyses				
WWI × Sex Interaction Model	WWI × Gender Term	0.029 (−0.119–0.177)	0.387	0.699

Note: Primary multivariable model is adjusted for age, sex, current smoking status, and BMI. Models in additional covariate analyses evaluate WWI (per 1 SD increase) after separately adding each indicated clinical covariate to the primary model. SIRI: Systemic Inflammation Response Index, WWI: Weight-adjusted Waist Index, CRP: C-reactive protein, eGFR: Estimated Glomerular Filtration Rate, CAD: Coronary Artery Disease.

## Data Availability

The data presented in this study are available on reasonable request from the corresponding author. The data are not publicly available due to privacy and ethical restrictions related to patient confidentiality.

## Supplementary Materials

The following supporting information can be downloaded at: https://www.mdpi.com/article/10.3390/metabo16100762/s1, Table S1: Formulas used to calculate anthropometric and CBC-derived systemic inflammatory indices; Table S2: Post-estimation regression diagnostics for the primary and secondary multivariable models; Figure S1: Post-estimation regression diagnostic plots for the primary multivariable linear regression model.

## Abbreviations

The following abbreviations are used in this manuscript:

MetS	Metabolic syndrome
BMI	Body mass index
WWI	Weight-adjusted waist index
BRI	Body roundness index
ABSI	A body shape index
WHR	Waist-to-hip ratio
WHtR	Waist-to-height ratio
SII	Systemic Immune-Inflammation Index
SIRI	Systemic Inflammation Response Index
NLR	Neutrophil-to-Lymphocyte Ratio
PLR	Platelet-to-Lymphocyte Ratio
LMR	Lymphocyte-to-monocyte ratio
CBC	Complete blood count
CRP	C-reactive protein
eGFR	Estimated glomerular filtration rate
FDR	False discovery rate
CAD	Coronary artery disease
