# Supplementary material for: Beyond BMI: Associations of Body-Shape Indices with CBC-Derived Systemic Inflammatory Indices in Adults with Metabolic Syndrome: A Cross-Sectional Study
Source: Metabolites. 2026 Oct 10;16(10):762. doi: 10.3390/metabo16100762 (PMC13706829; doi:10.3390/metabo16100762)
Supplement: Supplementary file 1 [file metabolites-16-00762-s001.zip › metabolites-4589093-supplementary.pdf]

Supplementary Table S1. Formulas used to calculate anthropometric and CBC-derived systemic inflammatory indices

Anthropometric Index	Formula
BMI	Weight (kg) / Height² (m²)
WHtR	Waist Circumference (cm) / Height (cm)
WWI	Waist Circumference (cm) / √Weight (kg)
BRI	364.2 − 365.5 × √{1 − [(WC / 2π)/(0.5 × Height)]²}
Conicity Index	Waist Circumference (m) / [0.109 × √(Weight (kg)/Height (m))]
ABSI	Waist Circumference (m) / [BMI^(2/3) × Height^(1/2)]
SIRI	(Neutrophil count × Monocyte count) / Lymphocyte count
SII	(Platelet count × Neutrophil count) / Lymphocyte count
NLR	Neutrophil count / Lymphocyte count
PLR	Platelet count / Lymphocyte count
LMR	Lymphocyte count / Monocyte count

BMI: Body Mass Index, *: kilogram/metre², WHtR: Waist-to-Height Ratio, WWI: Weight-Adjusted Waist Index, BRI: Body Roundness Index, ABSI: A Body Shape Index, SIRI: Systemic Inflammation Response Index, SII: Systemic Immune-inflammation Index, NLR: Neutrophil-to-lymphocyte Ratio, PLR: Platelet-to-lymphocyte Ratio, LMR: Lymphocyte-to-monocyte Ratio

Supplementary Table S2. Post-estimation regression diagnostics for the primary and secondary multivariable models

**Panel A. Multicollinearity diagnostics for the primary WWI–log-SIRI model**		
**Predictor**	**VIF**	**Tolerance**
Age	1.382	0.724
Sex	1.178	0.848
Current smoking	≤1.134	≥0.882
BMI	1.156	0.865
WWI	1.295	0.772

**Panel B. Model-level residual and influence diagnostics**					
**Outcome/model**	**Maximum Cook's D**	**|Std. residual| >3, n**	**Maximum |standardized residual|**	**Breusch–Pagan p**	**Durbin–Watson**
log-SIRI	0.062	2 (0.6%)	5.152	0.555	2.123
log-SII	0.071	3 (0.9%)	5.488	0.612	2.045
log-NLR	0.078	3 (0.9%)	4.479	0.489	2.087
log-PLR	0.055	3 (0.9%)	3.618	0.701	1.981
log-LMR	0.047	5 (1.5%)	3.717	0.534	2.016

Note: VIF, variance inflation factor; tolerance = 1/VIF. Smoking was coded as current versus non-current, with former smokers included in the non-current group. For the smoking term, the reported VIF and tolerance values represent conservative bounds based on the available diagnostic output. VIF values <2.5 were considered to indicate no problematic multicollinearity. Standardized residuals with an absolute value >3 were flagged as potentially unusual observations. Cook's distance was used to assess influential observations. The Breusch–Pagan test was used to evaluate heteroscedasticity, with p>0.05 indicating no statistical evidence of heteroscedasticity. Durbin–Watson statistics close to 2 were considered consistent with absence of substantial residual autocorrelation. Diagnostic measures were used to assess model adequacy and were not interpreted as hypothesis tests of the primary association.

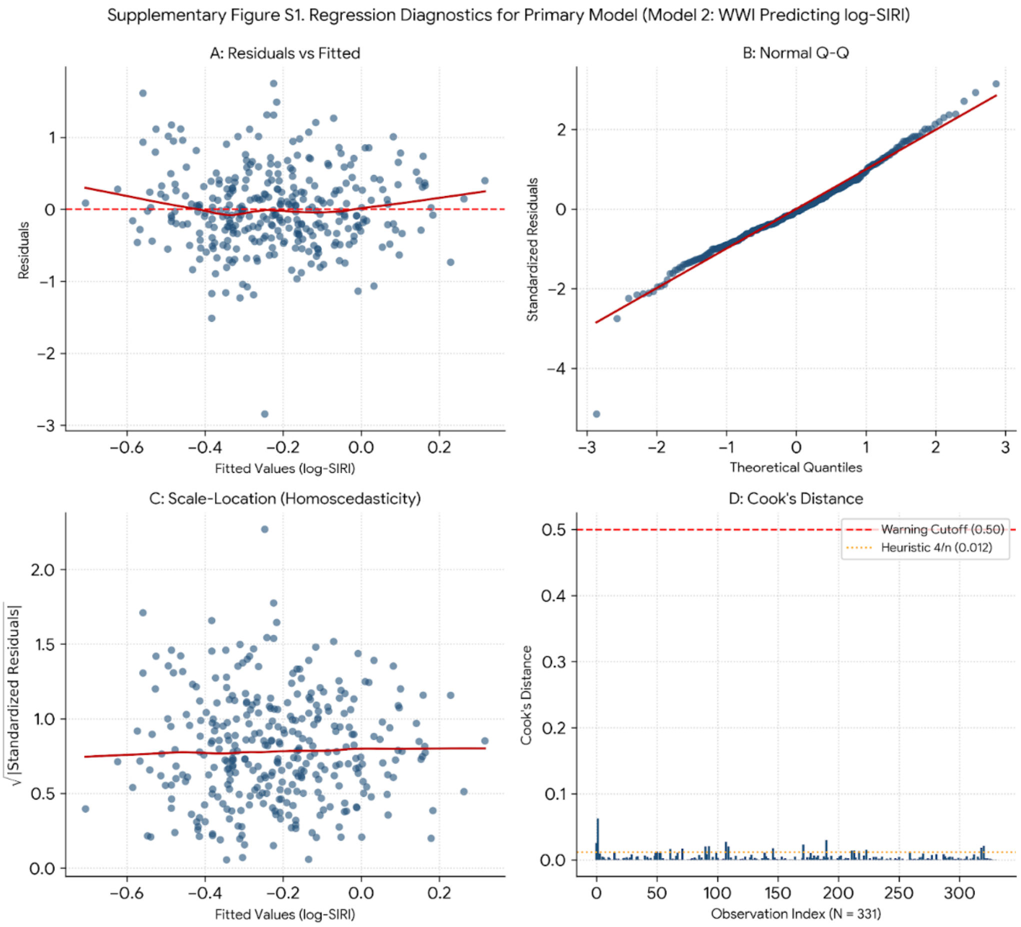

Supplementary Figure S1. Post-estimation regression diagnostic plots for the primary multivariable linear regression model (Model 2: WWI predicting log-SIRI).

(A) Residuals-versus-fitted plot showing no major systematic pattern in the residuals. (B) Normal Q–Q plot showing generally acceptable agreement with the theoretical quantiles, with some deviation in the tails. (C) Scale-location plot showing no clear increase in residual dispersion across fitted values; this was consistent with the Breusch–Pagan test (p=0.555), which provided no evidence of heteroscedasticity. (D) Cook's distance plot for all 331 participants. Some observations exceeded the heuristic 4/N threshold, but the maximum Cook's distance was 0.062 and no observation approached the 0.50 reference threshold. Thus, no single observation showed evidence of marked influence based on Cook's distance. Two observations had absolute standardized residuals >3; these observations were retained because their Cook's distance values did not indicate marked influence.
